# Electrochemical versus Photoelectrochemical Water Oxidation Kinetics on Bismuth Vanadate (Photo)anodes

**DOI:** 10.1021/jacs.4c03178

**Published:** 2024-04-25

**Authors:** Biwen Li, Louise I. Oldham, Lei Tian, Guanda Zhou, Shababa Selim, Ludmilla Steier, James R. Durrant

**Affiliations:** † Department of Chemistry, Centre for Processable Electronics, Imperial College London, London W12 0BZ, United Kingdom; ‡ Department of Materials and Environmental Chemistry, Stockholm University, Stockholm, SE-106 91, Sweden

## Abstract

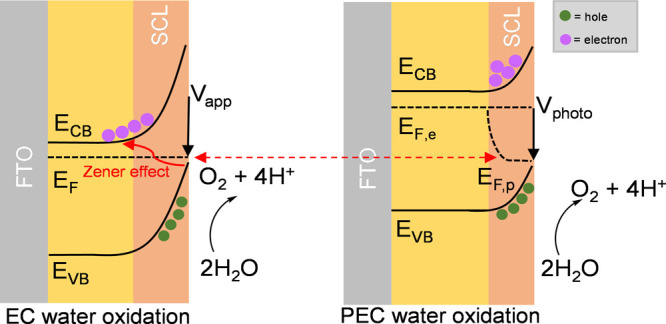

This study reports a comparison of the kinetics of electrochemical (EC) versus photoelectrochemical (PEC) water oxidation on bismuth vanadate (BiVO_4_) photoanodes. Plots of current density versus surface hole density, determined from operando optical absorption analyses under EC and PEC conditions, are found to be indistinguishable. We thus conclude that EC water oxidation is driven by the Zener effect tunneling electrons from the valence to conduction band under strong bias, with the kinetics of both EC and PEC water oxidation being determined by the density of accumulated surface valence band holes. We further demonstrate that our combined optical absorption/current density analyses enable an operando quantification of the BiVO_4_ photovoltage as a function of light intensity.

Metal oxides are extensively used for both photoelectrochemical (PEC) and electrochemical (EC) water oxidation.^1−3^ While it is widely recognized that the slow kinetics of water oxidation are a key limitation on the efficiency of both EC and PEC devices for green hydrogen synthesis, direct comparisons of EC versus PEC water oxidation kinetics have been limited to date. Different material classes are typically used in the EC and PEC systems, complicating such comparisons. Wide bandgap semiconducting metal oxides are typically used in PEC water oxidation, while more conductive, metallic metal oxides are typically used in EC systems. EC interfacial reaction kinetics are typically analyzed as a function of applied potential, with the potential modulating the reaction activation energy.^4,5^ For PEC reactions, a complication in such analyses is the additional photovoltage generated by irradiation, which can be difficult to quantify under operando conditions.^6^ An additional consideration is that several recent studies, including our own, have highlighted the importance of the chemical rather than potential driven rate-determining steps for both EC and PEC water oxidation catalysis.^7,8^ In this case, the reaction kinetics are primarily determined not by the potential but by the population density of the reactive intermediates. In the study herein, we focus on operando experimental determination of such population densities for both EC and PEC water oxidation on a widely studied metal oxide, bismuth vanadate (BiVO_4_), enabling both a direct comparison of EC and PEC reaction kinetics and operando quantification of the photovoltage generated under light irradiation.

BiVO_4_ is a semiconductor with a bandgap of 2.4 eV and is typically n-doped due to the presence of oxygen vacancies. For single-electron outer-sphere reactions, different reaction mechanisms are typically observed for EC and PEC reactions on semiconducting electrodes. For n-type semiconductors, such EC oxidation reactions are normally driven by electron tunnelling from reactants in the electrolyte into the semiconductor conduction band (CB), without direct involvement of the semiconductor valence band (VB).^9−15^ In contrast, PEC oxidation reactions are normally considered to be driven by VB holes generated by optical excitation of electrons into the CB.^16^ However, it is not clear if this difference in reaction mechanism is relevant to multiredox reactions such as water oxidation studied herein. The PEC reaction mechanism can be understood thermodynamically by light irradiation, resulting in a splitting of the electron and hole quasi-Fermi levels (*E*_*F,n*_ and *E*_*F,p*_, respectively) by the photovoltage *V*_*photo,*_. The simplest assays of *V*_*photo*_ derive from comparison of light and dark current–voltage (*J*–*V*) curves and onset potentials,^17^ while surface photovoltage measurements provide an alternative assay.^18−20^ However, such measurements do not provide direct assays of the underlying Fermi level splitting, complicating their interpretation. In the study herein, we address these uncertainties as well as addressing whether a population density model based on a chemical rate-determining step is applicable to both EC and PEC water oxidation on BiVO_4._

BiVO_4_ (photo)anodes were fabricated using a modified metal organic deposition method (details in ESI).^21^ Cyclic voltammetry was used to confirm that the BiVO_4_ films were dense and pinhole free (Figures S1 and S15),^22^ to exclude possible involvement of any exposed fluorine-doped tin oxide substrate during EC water oxidation. To examine the PEC and EC performance, the *J*–*V* characteristics of BiVO_4_ (photo)anodes were measured under chopped simulated AM 1.5 light and in the dark (Figure 1) in a three-electrode PEC cell. It is apparent that the photocurrent onset is shifted cathodically by ca. 1.7 V relative to the dark current, attributed to the photovoltage generated under irradiation.

**Figure 1:**
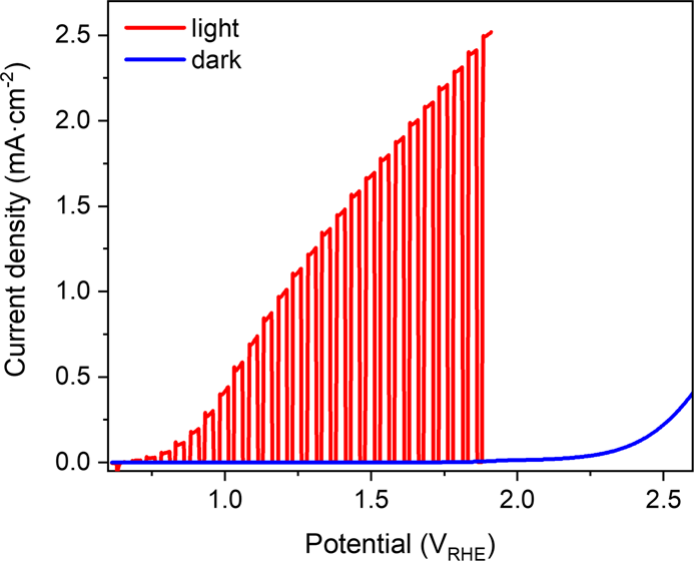
Current–voltage characteristics of BiVO_4_ under chopped light (one sun equivalent) and dark conditions.

To elucidate the underlying difference in light and dark *J*–*V* curves, photoinduced absorption (PIA) and stepped potential spectroelectrochemistry (SP-SEC) were used to evaluate the operando optical absorption changes during PEC and EC water oxidation, respectively. Both techniques apply a square wave pump signal to perturb steady state water oxidation. In PIA, the pump signal was provided by a variable intensity 365 nm LED light at a fixed potential, in SP-SEC by a variable voltage square wave potential, as shown in Figure 2 inset (see the Supporting Information for details).^8,23^ In both cases, the resultant optical absorption and current transients were employed to quantify the population densities and reaction kinetics.

**Figure 2:**
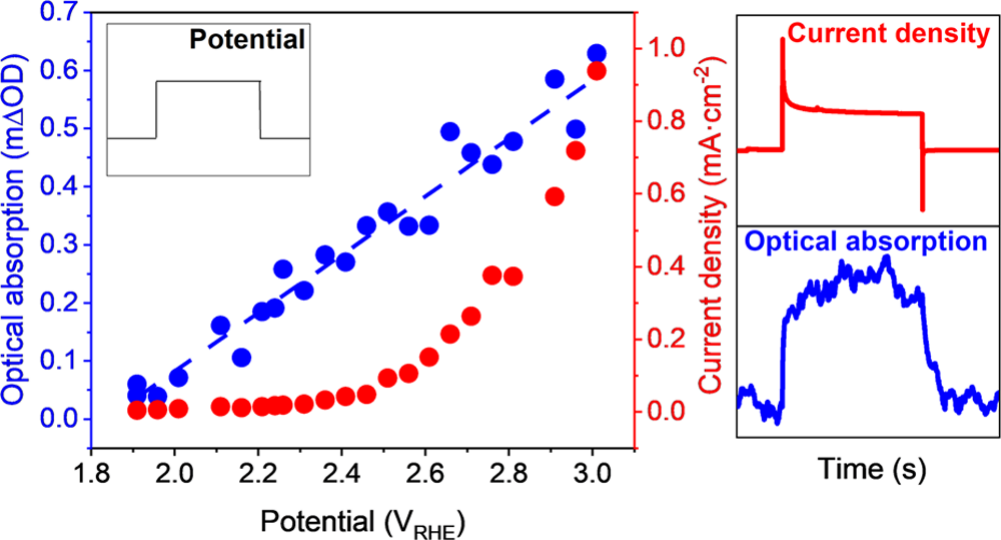
SP-SEC measurements. Optical absorption (550 nm) and current recorded synchronously under each potential step (inset) are shown against applied potential. Right panel: current density and optical absorption trace at a specific applied potential.

We have previously reported PIA studies of water oxidation on similar BiVO_4_ photoanodes, employing a probe wavelength of 550 nm assigned to the absorbance of photogenerated surface VB holes.^21^ These measurements were extended herein to dark EC water oxidation, again employing a 550 nm probe (Figure 2, see also Figure S4 for equivalent PIA under light irradiation). It is apparent that the 550 nm absorbance correlates with the EC water oxidation current, indicative of this dark water oxidation also being driven by the VB holes. Full spectral data as a function of applied bias is shown in Figure S2. In addition to a broad absorption from 550 to 800 nm assigned to surface holes, a narrow absorption peak was also observed centered at 460 nm. This 460 nm absorption appeared at much lower applied potentials than the onset of water oxidation (Figure S3) and is therefore assigned, as previously, to intragap states unable to drive water oxidation.^24^ It is apparent from Figure 2 that the 550 nm optical VB hole signal increases approximately linearly with applied potential, while *J*_*wo*_ increases nonlinearly, as we analyze further below.

We turn now to a comparison of EC and PEC water oxidation kinetics. The hole absorption at 550 nm can be converted to a surface hole density *p*_*s*_ using the Beer–Lambert law with a molar extinction coefficient of 420 M^–1^cm^–1^.^25^ Further, the measured current density *J*_*wo*_ divided by *p*_*s*_ gives the reaction turnover frequency (TOF) per surface hole. Figure 3 shows plots of *J*_*wo*_ and TOF versus *p*_*s*_ for both EC and PEC water oxidation on BiVO_4_. For both measurements, biphasic behavior is observed. Applying a rate law model, as we have applied previously to PEC water oxidation on BiVO_4_,^25^ our EC data demonstrate first-order behavior (α = 0.95 ± 0.11) in the low *p*_*s*_ region (most likely resulting from hydroxide oxidation to hydroxyl radicals), explaining the low current <2.4 V_RHE_.^8,26^ At higher hole densities, third-order behavior is observed (α = 2.95 ± 0.26), assigned to water oxidation to molecular oxygen.^25^ Most strikingly, while plots of *J*_*wo*_ versus potential are very different in the dark and light (see Figure 1), our plots of *J*_*wo*_ vs *p*_*s*_ are indistinguishable between the EC and PEC data within experimental error (see Figure S9 for comparison of data from a further electrode). Such overlapping kinetic behavior was also found from PEC and EC optical decays (Figure S11). An analogous observation has been reported recently by Cowan et al. for hematite photoanodes.^27^ An overlap is also observed in our plot of the TOF versus *p*_*s*_ (Figure 3b). This indicates that the kinetics of water oxidation in the light and dark, when measured at matched surface hole densities, are indistinguishable. This overlap provides clear evidence that dark EC and light PEC water oxidations on BiVO_4_ have equivalent reaction mechanisms and that both are driven by the accumulation of surface VB holes.

**Figure 3:**
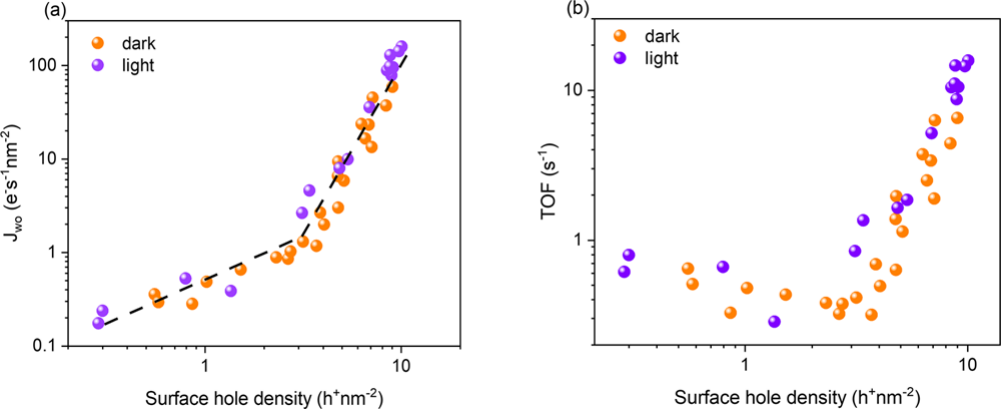
(a) The steady state (photo)current densities *J*_wo_ and (b) TOF under PEC (purple) and EC (orange) conditions versus *p*_s_.

Our conclusion that the kinetics of both EC and PEC water oxidation are determined only by the density of VB holes rather than the applied potential is consistent with several recent reports from other groups.^7,28^ It confirms that EC water oxidation on BiVO_4_ is not driven by electron injection into the BiVO_4_ CB, in contrast to previously studied EC oxidation reactions on n-type semiconductors.^9−15^ Our observation of VB hole driven EC water oxidation suggests an alternative mechanism, as illustrated in Scheme 1, where the strong electric fields present in the space charge layer (SCL) under EC water oxidation conditions can promote electron tunnelling from the VB at the surface into the bulk CB through the so-called internal field emission, or Zener effect.^15,29,30^ This Zener effect is enabled by the narrow width of the SCL (∼50 Å at 3.2 V_RHE_, see Figure S5) and the magnitude of the band bending (the onset dark potential, ca. 2.4 V_RHE_ and the flatband potential, 0.35 V_RHE_) being comparable to the bandgap of BiVO_4_.^15^ Following this model, the main difference between PEC and EC water oxidation is the method of VB hole generation, with this being driven by the high SCL electric fields in the dark and by photoexcitation in the light. This difference from classical theory most likely originates from the chemical rather than potential driven nature of the rate-determining step for water oxidation on BiVO_4_, which is associated with this being a multiredox, inner-sphere reaction. It furthermore indicates that the underlying mechanism of water oxidation catalysis on BiVO_4_ is the same for both EC and PEC conditions, independent of the magnitude of the applied potential and potential drops across the Helmholtz layer.

**Scheme 1:**
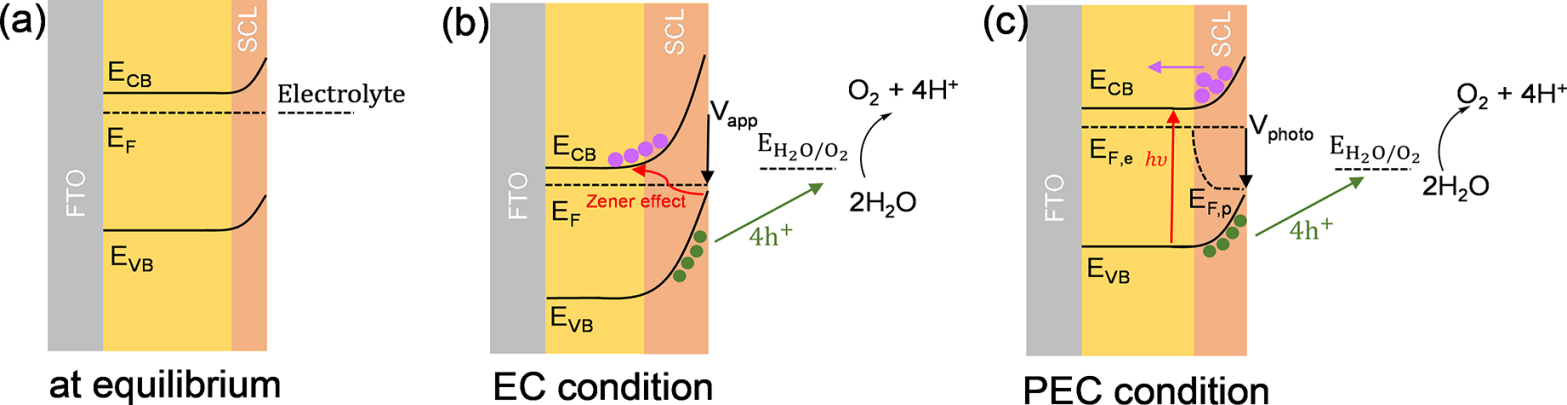
Band diagrams of BiVO_4_ (a) Dark; (b) dark, under a strong anodic bias; (c) light, under a smaller anodic bias. In the dark, band bending increases with *V*_*app*_ (panel b). In the light, *E*_*F*_ splits into *E*_*F,e*_ and *E*_*F,p*_ (panel c). Holes (green dots) accumulated in the SCL when strong anodic potentials or light are applied, and electrons (purple dots) drifted away from the SCL.

Having established the equivalent population driven mechanisms for both EC and PEC water oxidation, we can employ our PIA and SP-SEC data to obtain an operando determination of *V*_*photo*_, as a function of the light intensity, ϕ. In our EC measurement, the applied potential *V*_*app*_ can be taken as a measure of the BiVO_4_ surface *E*_*F*_, assuming minimal resistance losses. At matched surface hole densities, this dark *E*_*F*_(*p*_*s*_, dark) value will be equivalent to the *E*_*F,p*_(*p*_*s*_, light) present under irradiation (Scheme 1). As such, *V*_*photo*_ can be most simply determined from *V*_*photo*_(*p*_*s*_) *= E*_*F*_(*p*_*s*_, dark)/e – *V*_*app*_(light), where for the studies herein *V*_*app*_(light) was held at 1.7 V_RHE_. In Figure 4 (a), the *V*_*photo*_ is plotted versus ϕ, illustrating a logarithmic dependence () (see Figure S13 for further electrodes). We also determined *V*_*photo*_ using the difference in light and dark onset potentials (absence of significant current flow and so not properly operando) and observed qualitatively similar behavior (Figure S14a and b). For both data sets, “nonideal” behavior is observed (*n* > 2), as has also been reported for hematite and SrTiO_3_ photoanodes.^17,20^ The origin of this nonideal behavior is not clear but most likely associated with the impact of intragap defect states and/or “photocharging” effects widely reported for BiVO_4_.^31−35^ A full analysis of this nonideal behavior is beyond the scope of this study. Figure 4 (b) shows a plot of the *J*_*wo*_ of EC and PEC versus *E*_*F,p*_, where we use in the dark *E*_*F,p*_ = *V*_*app*_(dark) and in the light *E*_*F,p*_ = *V*_*app*_(light) + *V*_*photo*_. It is apparent that these two plots of the current density versus *E*_*F,p*_ overlap, strongly supporting the validity of our determination of *V*_*photo*_ and emphasizing that the water oxidation current is determined only by the position of *E*_*F,p*_, and therefore *p*_*s*_, independent of whether the hole population is generated electrochemically or photoelectrochemically. In contrast to other approaches to determine the photovoltage,^17−20^ the experimental approach demonstrated herein provides an operando determination of hole quasi-Fermi level as a function of light intensity, opening up the potential for in depth analyses of photovoltage generation.

**Figure 4:**
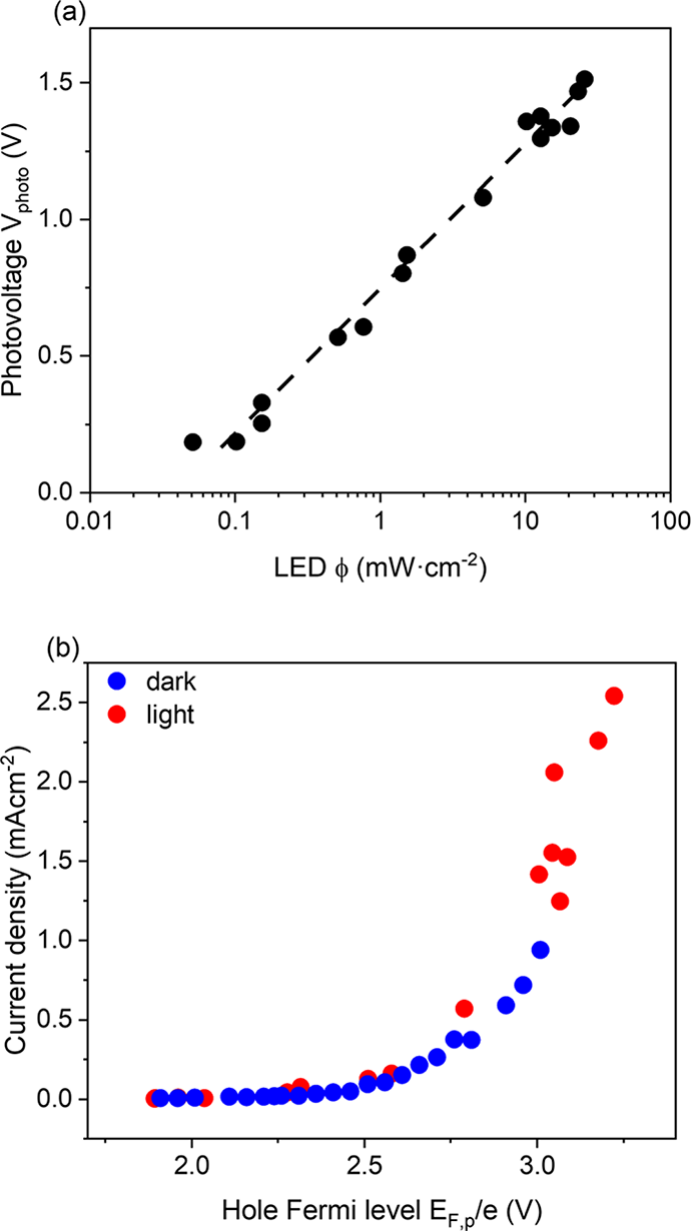
(a) Relationship between *V*_*photo*_ and ϕ under PEC conditions. (b) The overlap of dark and light current density versus *E*_*F,p*_.

To summarize, we have demonstrated that water oxidation on BiVO_4_ proceeds via the same kinetics and mechanism under PEC and EC conditions. The accumulation of a sufficient density of surface VB holes is shown to be the key step for both PEC and EC water oxidation, consistent with a chemical rather than potential driven rate determining step. Moreover, the optical absorption-based analyses enable the operando determination of *V*_*photo*_ as a function of light irradiation. While further study is needed to determine the wider applicability of this methodology to determine *V*_*photo*_, such as the potential impact of Fermi level pinning, it offers a new approach to investigating operando photovoltage generation in photoelectrodes.

## Supporting Information Available

Additional information including BiVO_4_ preparations, experiment procedures (LSV, CV and operando PIA and SP-SEC), and detailed explanation mentioned in main text (PDF)
