# Supplementary material for: Electrochemical versus Photoelectrochemical Water Oxidation Kinetics on Bismuth Vanadate (Photo)anodes
Source: J Am Chem Soc. 2024 Apr 25;146(18):12324–8. doi: 10.1021/jacs.4c03178 (PMC11082883; doi:10.1021/jacs.4c03178)
Supplement: Supplementary file 1 — ja4c03178_si_001.pdf [file ja4c03178_si_001.pdf]

Supporting information for

Electrochemical versus Photoelectrochemical Water Oxidation Kinetics on Bismuth Vanadate (Photo)anodes

Biwen Li,[†,§] Louise I. Oldham,[†] Lei Tian,[*,†,‡] Guanda Zhou,[†,∥] Shababa Selim,[†] Ludmilla Steier,[†,⊥] and James R. Durrant[*,†]

[†]Department of Chemistry, Centre for Processable Electronics, Imperial College London, London W12 0BZ, U.K.
[‡]Department of Materials and Environmental Chemistry, Stockholm University, Stockholm, SE-106 91, Sweden.

1. Experimental methods

1.1 $BiVO_4$ electrode fabrication

All chemicals were purchased from Sigma-Aldrich and used without further purification. $BiVO_4$ electrodes were fabricated by modified metal-organic deposition reported by Ma *et al.*[1] To prepare $BiVO_4$ solution for spin coating, bismuth nitrate pentahydrate (0.1455 g, 0.30 mmol) was dissolved in acetic acid (1.50 mL). The vanadyl acetylacetonate powder (0.0768 g, 0.29 mmol) was dissolved in acetylacetone (5.00 mL). Subsequently, the vanadyl acetylacetonate solution was added to the bismuth nitrate solution and washed with acetylacetone (5.00 mL in total). The mixture was stirred at room temperature for 25 minutes to form a turquoise sol-gel. Fluorine-doped tin oxide (FTO) glass pieces (5.0 × 5.0 cm) were cleaned by sonication for 15 mins in detergent solution, de-ionized water, acetone, and isopropanol, respectively. After drying, the FTO glass was cleaned with ozone for 10 minutes. To prepare the $BiVO_4$ photoanode film, 14 layers of solution were deposited on FTO glass. For the first layer, the sol-gel (0.25 mL) was spin-coated (Model WS-400A-6Npp/LITE) to the glass at a spin rate of 2000 rpm for 20 seconds, calcined at 500 ℃ for 10 minutes, then cooled at room temperature for 10 minutes. For the remaining 13 layers, the sol-gel (0.25 mL) was spin-coated at a spin rate of 1000 rpm for 20 seconds. Following the spin coating of each layer, the films were calcined and

cooled as above with the exception of the final layer, which was calcined at 500 ℃ for five hours before being cooled to room temperature.

1.2 Photochemical (PEC) and electrochemical (EC) characterization

The films were characterized by linear sweep voltammetry and cyclic voltammetry (CV). All EC characterization was carried out in a home-made three-electrode EC cell with a 0.5 cm^2 quartz window. A Ag/AgCl electrode in saturated KCl solution and a platinum mesh were used as the reference and counter electrodes, respectively. All the applied potentials in experiments were with reference to Ag/AgCl (sat. KCl) electrode, and were converted to the reversible hydrogen electrode (V_{RHE}) by Equation S1.

$$V_{RHE} = V + 0.059 \times pH + 0.198(\text{Ag/AgCl}) \quad \text{(S1)}$$

where V is the applied potential in the experiment, and 0.198 V is the standard reduction potential of Ag/AgCl electrode in saturated KCl solution.

$BiVO_4$ films were used as the working electrode. The linear sweep voltammetry was measured in potassium phosphate aqueous buffer (KPi, 0.1 M, pH = 7), to characterize its J-V performance of $BiVO_4$ films. For PEC characterization, the excitation light was provided by a 365 nm LED source and the sample was illuminated from the front (i.e. the side of the $BiVO_4$/electrolyte interface). The light intensity was 12.6 $mWcm^{-2}$ (equivalent to one sun (AM 1.5)) measured by a power meter.

Based on a reported method, CV was used to confirm that the $BiVO_4$ films were pinhole-free, namely that the underlying FTO was not participating in EC water oxidation.[2] CV measurements were carried out in the presence of $K_4Fe(CN)_6$ and $K_3Fe(CN)_6$ redox couple (1 mM , pH = 5.95). As shown in Figure S1 (left panel), there were pronounced redox peaks with bare FTO. The anodic peak and cathodic peak centered at 0.95 and 0.77 V_{RHE}, respectively. During oxidation, electrons

transfer from $[Fe(CN)_6]^{2+}$ ions to FTO generating $[Fe(CN)_6]^{3+}$. During reduction, electrons transfer from FTO to $[Fe(CN)_6]^{3+}$ ions, generating $[Fe(CN)_6]^{2+}$. $[Fe(CN)_6]^{3+}/[Fe(CN)_6]^{2+}$ was chosen as the redox couple because its reduction potential (estimated with $E_{1/2}$ =0.86 V_{RHE}) is in the $BiVO_4$ bandgap. This means no redox peaks should be observed if a dense $BiVO_4$ layer was deposited on FTO. As shown in Figure S1 (left panel), no clear anodic and cathodic peaks were observed for the $BiVO_4$/FTO anode (there may be some small current contributions from FTO (e.g. at the edges) in the sample of FTO_$BiVO_4$, but it was negligible compared to the current observed in the FTO sample), indicating electron transfers between FTO and the redox species were indeed prohibited by the $BiVO_4$ top layer. To further consolidate our conclusion, the $[Ru(bpy)_3]^{3+/2+}$ redox couple (pH = 3.0) with a more positive reduction potential was also used to avoid possible influence from deep trap states below the CB of $BiVO_4$. The reduction potential of $[Ru(bpy)_3]^{3+/2+}$ can be estimated to be $E_{1/2}$ = 1.63 V_{RHE} which is more positive than $E_{1/2}$ = 0.86 V_{RHE} of $[Fe(CN)_6]^{3+/2+}$, so the reduction potential of $[Ru(bpy)_3]^{3+/2+}$ is located deeper into the bandgap of $BiVO_4$ than that of $[Fe(CN)_6]^{3+/2+}$. Like the analysis above, from Figure S1 (right panel), we can further conclude that spin-coated $BiVO_4$ can be treated as pinhole-free.

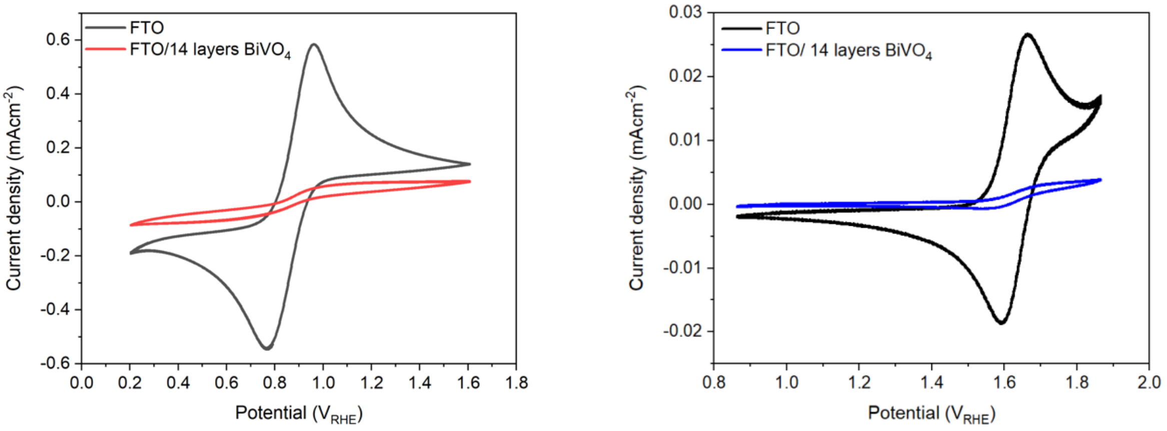

Figure S1. Cyclic voltammetry of FTO and $BiVO_4$/FTO (with 14 layers of $BiVO_4$) using $[Fe(CN)_6]^{3+}/[Fe(CN)_6]^{2+}$ redox couple ((1 mM, pH = 5.95), left panel) and

$[Ru(bpy)_3]^{3+}/[Ru(bpy)_3]^{2+}$ redox couple ((0.1 mM, pH = 3.0), right panel).

1.3 Photo-induced absorption (PIA) spectroscopy

PIA measurements were carried out on a home-built system which simultaneously recorded the optical absorption, corresponding to $BiVO_4$ hole absorption, and photocurrent during PEC water oxidation. A 365 nm LED was used to generate a square wave pump light (10 s on_5 s off). Surface hole density was modulated by changing the LED intensity, controlled through the power output of the LED power supply (Aim-TTi QL355).

The probe light source was a tungsten halogen lamp (OSRAM 64640 HLX, Bentham IL1 lamp housing, Bentham 605 power supply) and selected for 550 nm by a monochromator (Oriel Cornerstone 130 1/8 m) placed after the sample. Long pass filters (515 nm and 530 nm) were placed before the sample to minimize excitation of the sample by the probe light, and a color wheel (Thorlabs FW102C) was placed between the sample and the monochromator. The probe light was focused/collimated by a series of optical lenses and mirrors. The sample itself was in the three-electrode cell setup described above. An Autolab potentiostat (Metrohm PGSTAT 101) was used to hold the sample at 1.7 V_{RHE} throughout the PIA measurements. This potential was selected to ensure back electron recombination was minimized.

The transmittance of photons through the sample was detected by a silicon diode detector (Costronics 2002) and converted into a potential signal which was recorded by a National Instruments DAQ card (NI USB-6251). The potential signal was also amplified (Costronics 2008) and then recorded by an oscilloscope (Tektronics TPO 3012). The recorded signals from the DAQ card were the average of 5 to 20 pump pulses.

The photocurrent was measured as a change in potential across a fixed resistor inside the potentiostat. The potential signal was recorded by a separate channel in the oscilloscope and converted to a photocurrent using Ohm's Law. The whole system was controlled through a bespoke Labview programme, which was used to command the DAQ card to trigger the oscilloscope, begin data acquisition and turn on/off the LED pump via a MOSFET (ZVN 430 6A) switch.

1.4 Operando step-potential spectroelectrochemistry (SP-SEC)

SP-SEC used the same experimental setup as PIA but the LED pump source was replaced with a square wave potential. The potential pump was provided by the Autolab potentiostat (Metrohm PGSTAT 101), controlled through NOVA software and set up as follows:

V_0 = 0.61 V_{RHE}, applied for 2 s; V_1 was an adjustable parameter to modulate the hole population, applied for 8 s to reach steady state; V_2 = 0.61 V_{RHE}, applied for 5 s to allow the system to relax to steady state. For the measurement of the signal decay in open circuit conditions, the system was switched to open circuit after V_1 instead of applying V_2 = 0.61 V_{RHE}.

The value of V_0 was chosen to be 0.61 V_{RHE} because this was the initial measured value of the open circuit potential (OCP). The OCP value was recorded before running any EC experiments when the three-electrode cell was set, namely all three electrodes were in contact with the KPi buffer (pH = 7) and with electrical connection to the potentiostat.

2. The potential-induced optical absorption spectrum of $BiVO_4$ anode under SP-SEC characterization

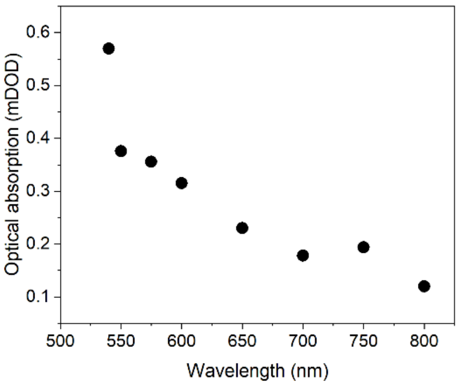

Figure S2. The potential-induced optical absorption of $BiVO_4$ anode under EC water oxidation conditions. Data collected across a spectral range from 540 nm to 800 nm under a square wave pump potential of V_0 = 0.61 V_{RHE}, V_1 = 2.9 V_{RHE}, and V_2 = 0.61 V_{RHE}.

Under EC conditions, we did not observe an absorption peak at ~550 nm as reported under PEC conditions.[1] Instead, the optical absorption decreased from 540 nm to 800 nm. The disappearance of the 550 nm peak could be due to extra photoinduced absorption contributed from the probe light. If the wavelength of the probe light is set below 550 nm (e.g. 540 nm), its photon energy is approaching the bandgap of $BiVO_4$ (2.4 eV). Once $BiVO_4$ is photo-excited, photo-generated holes will accumulate on the VB of $BiVO_4$, and the optical absorption of photo-generated holes will be superimposed on that of the potential-induced holes.

3. The optical absorption of $BiVO_4$ at 460 nm under SP-SEC characterization

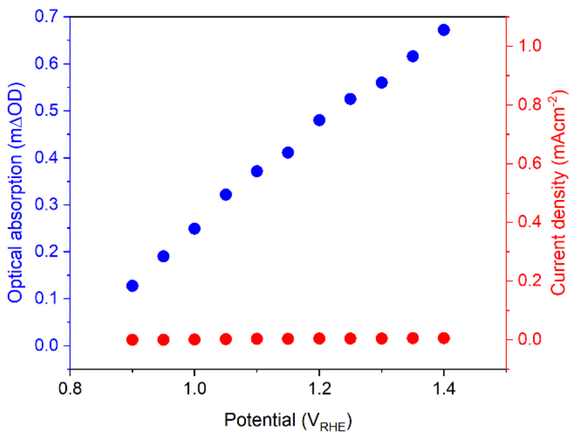

Figure S3. The 460 nm optical absorption and current density of $BiVO_4$ plotted as a function of applied potential.

The optical absorption at 460 nm increased with the potential, but there was no water oxidation current observed, even when an optical absorption was up to 0.7 mΔOD. Thus, the optical signal at 460 nm is not related to the water oxidation.

4. The dependence of PIA signal and photocurrent density on LED intensity.

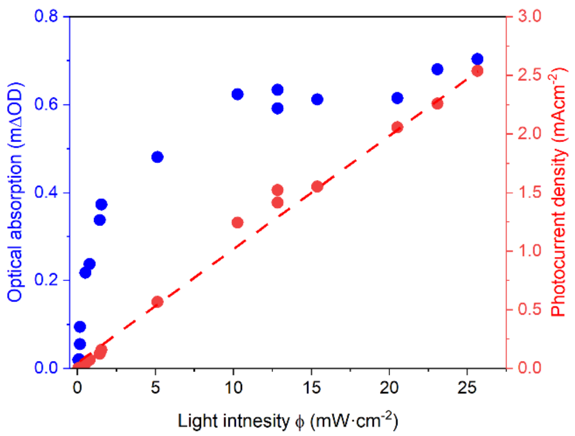

Figure S4. Steady state PIA signals at 550 nm (blue) and photocurrent density (red) as a function of LED intensity.

5. The dependence of optical absorption at 550 nm, space charge layer (SCL) width and electric field strength on potential.

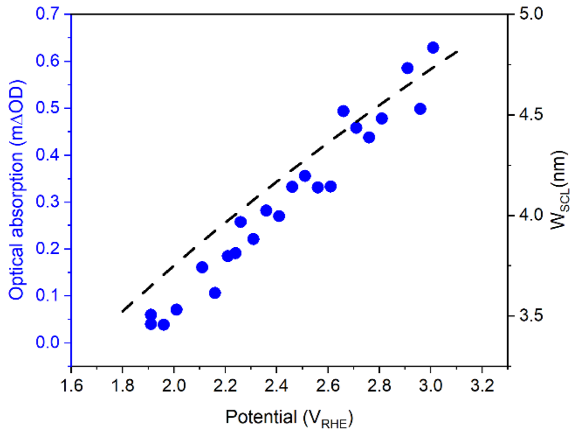

Figure S5. Optical absorption at 550 nm and the width of the space charge layer plotted as a function of applied potential.

The SCL width was calculated using the equation S2:

$$W_{SCL} = \sqrt{\frac{2\varepsilon_0\varepsilon(V - V_{FB})}{eN_D}} \qquad \text{(S2)}$$

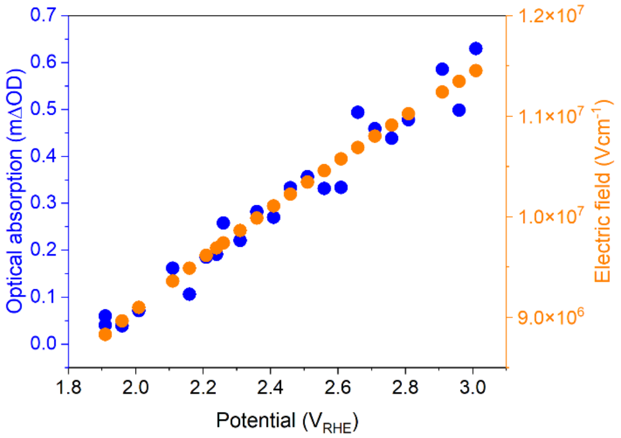

Figure S6. Optical absorption at 550 nm and the electric field strength plotted as a function of applied potential.

The electric field strength was calculated using the equation S3:

$$E = \frac{eN_D W_{SCL}}{\varepsilon\varepsilon_0} \qquad \text{(S3)}$$

where ε_0 is vacuum permittivity, ε is relative permittivity of $BiVO_4$ (ε = 68)[3], V is applied potential in experiments, V_{FB} is flat band potential (0.35 V_{RHE}), e is elementary charge and N_D is donor density (9.1×10^{20} cm^{-3}) in $BiVO_4$. The values of V_{FB} and N_D are quoted from reference.[3]

Figures S5 and S6 show how the hole signal in the dark correlates with the width of the SCL and the electric field strength respectively. The width of the SCL and the electric field strength both increase with applied potential, following a similar trend to the optical absorption of surface holes.

6. The kinetic analysis of water oxidation on $BiVO_4$.

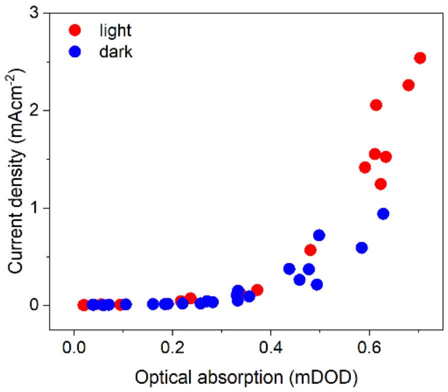

Figure S7. The overlay of $BiVO_4$ current density and optical absorption plots under light and dark conditions, plotted on a linear-linear scale.

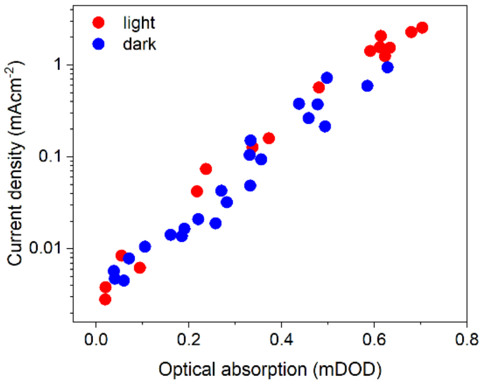

Figure S8. The overlay of $BiVO_4$ current density and optical absorption plots under light and dark conditions, plotted on a log-linear scale.

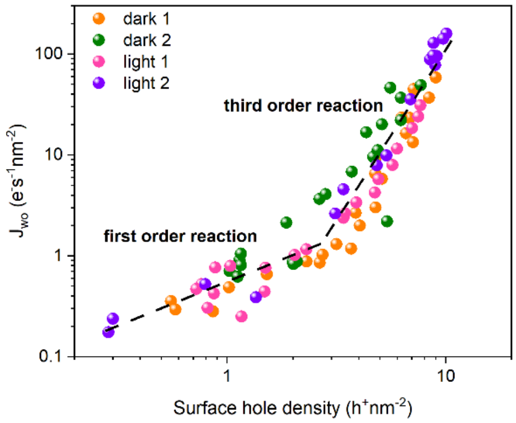

Figure S9. The rate law plots of water oxidation on different $BiVO_4$ electrodes under PEC and EC conditions. The PIA and SP-SEC measurements were further repeated to show the reproducibility of the overlay kinetics of PEC and EC water oxidation on $BiVO_4$ (the datasets from Dark1 and Light1 were collected from an identical $BiVO_4$ film; the datasets from Dark2 and Light2 were collected from different $BiVO_4$ films).

Table S1. A summary of orders of water oxidation in surface holes of different $BiVO_4$ films under PEC and EC conditions.

	α (low surface hole density)	α (high surface hole density)
Dark 1	0.95 ± 0.11	2.95 ± 0.26
Dark 2	1.00 ± 0.25	2.73 ± 0.63
Light 1	1.06 ± 0.22	2.99 ± 0.20
Light 2	0.95 ± 0.24	3.13 ± 0.16

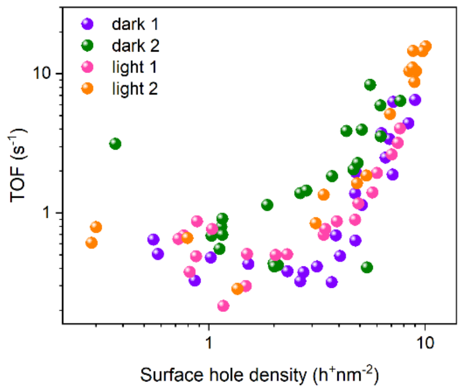

Figure S10. A log-log plot of the turnover frequency (TOF) of $BiVO_4$ holes for water oxidation with respect to the surface hole density under PEC and EC conditions.

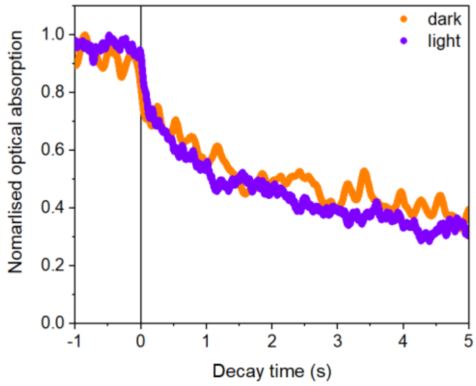

Figure S11. The overlays of PEC and EC water oxidation optical decay transients when the LED was switched off (light, purple) and the system was switched to open circuit (dark, orange). The only pathway to consume the surface holes generated is *via* water oxidation. The decay starts at decay time = 0s.

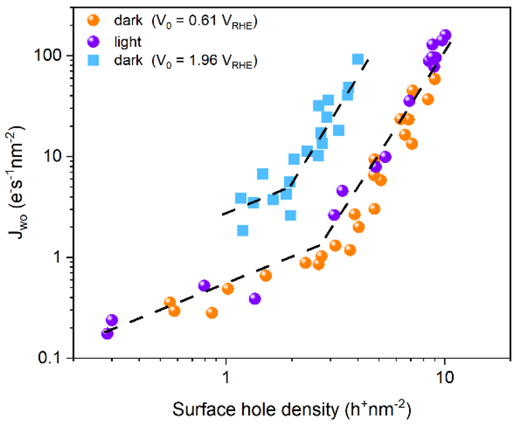

Figure S12. A comparison of rate law using different V_0 values under SP-SEC characterization.

To understand the effect of V_0 on rate law analysis, the dark rate law was also measured using V_0 at 1.96 V_{RHE}. As shown in Figure S12, although two linear regions seem observed, for a given current density the surface hole density (i.e. optical absorption of holes) at V_0 = 1.96 V_{RHE} is smaller compared to V_0 = 0.61 V_{RHE}. In SP-SEC measurements, the optical absorption of surface holes at 550 nm is equal to the optical absorption when V_1 is applied minus the optical absorption when V_0 is applied (i.e. reference) (equation S4).

$$\Delta OD = OD_{V1} - OD_{V0} \qquad \text{(S4)}$$

It is possible for potential-induced holes to be generated when V_{app} is anodic of V_{OCP}. Therefore, when V_0 was set to 0.61 V_{RHE} (initial measured V_{OCP}), all the potential-induced holes were measured. However, when V_0 was set to 1.9 V_{RHE}, the hole signal at this potential was used as the reference, and the measured hole signal therefore did not include any holes generated between 0.61 V_{RHE} and 1.9 V_{RHE}. This is the likely explanation for the shifted dark J_{wo} *vs*. p_s curves for the two V_0 values.

7. Comparisons of photovoltage calculated using two different methods.

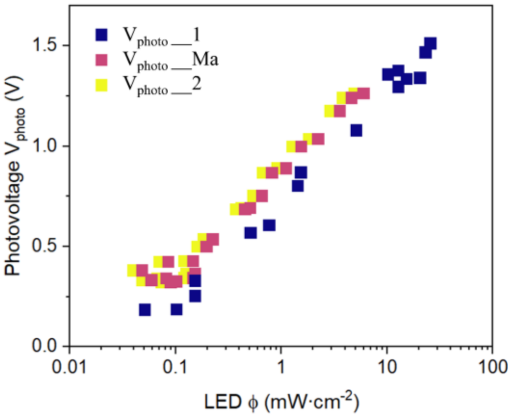

Figure S13. The repeatability comparison of photovoltage versus LED intensity (ϕ) with data collected from multiple samples, calculated from the operando photovoltage method used in Figure 4(a). For V_{photo}_1, the data from Figure 4(a) were added for comparison. For V_{photo}_2, the PIA data (optical absorption versus light intensity) were collected using a new $BiVO_4$ sample (i.e. the same PIA dataset used in Light2 in Figure S9), and the SP-SEC data (optical absorption versus applied potential) were collected from a further $BiVO_4$ sample (i.e. the same SP-SEC dataset used in Dark2 in Figure S9). For V_{photo}_Ma, the PIA data were directly adapted from a reported work from Ma *et.al*,[1] and the SP-SEC data were from the dataset used in Dark2.

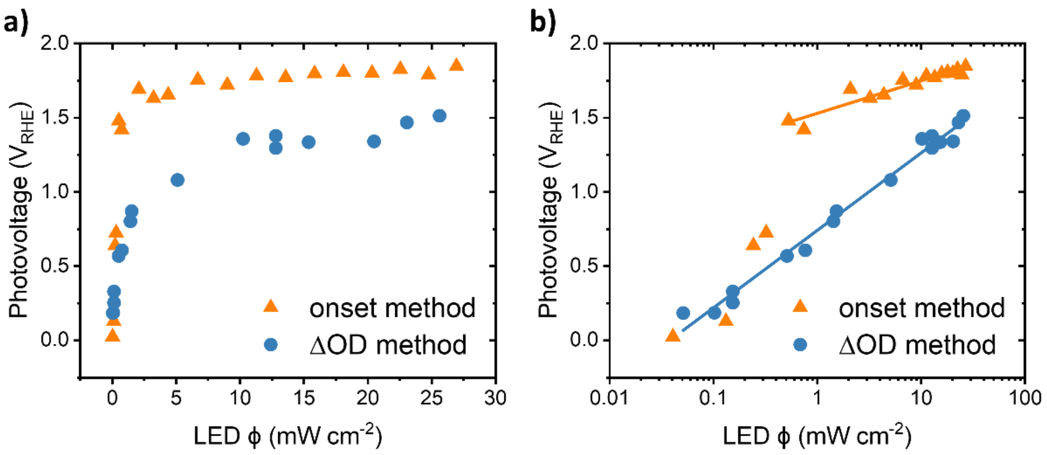

Figure S14. Light intensity-dependence of photovoltage in $BiVO_4$ photoanodes, derived from the onset potentials method (orange, triangle) and the operando ΔOD method (blue, circle). LED intensity (ϕ) plotted on (a) a linear scale and (b) a $\log_{10}$ scale. For the onset potential method, the (photo)current threshold was defined as 0.02 mAcm^{-2}.

Photovoltage can be calculated by taking the difference in (photo)current onset potentials in the light and the dark from the J-V curves. Figure S14 compares the light intensity-dependence of photovoltage calculated by this onset method with the photovoltage calculated by the operando ΔOD method described in the main text.

When plotted on a linear LED (ϕ) scale (Figure S14a), the photovoltage begins to saturate at higher light intensities when measured by both methods. When plotted on a logarithmic LED (ϕ) scale (Figure S14b), photovoltage from both methods demonstrates linear dependence on the log of light intensity at higher light intensities. The data from both methods show a photovoltage with non-ideal behavior, although this is much stronger in the data from the ΔOD method compared to the onset method. Notably, when measured from the onset method, the photovoltage drops off rapidly at low light intensities whereas the photovoltage from the ΔOD method does not deviate from its linear behavior. A detailed comparison of these two photovoltage methods and their meaning is beyond the scope of this paper but is the subject of ongoing study.

8. Scanning microscopic image (SEM) of $BiVO_4$ film.

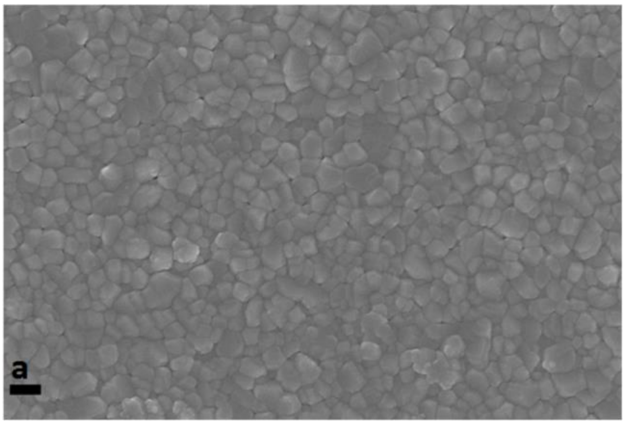

Figure S15. A top-view SEM image of $BiVO_4$ films synthesized by the same method used in this paper, showing a dense structure packing by ~100 nm particles (the scale bar is 200 nm). Reproduced from Ref. 4 with permission from the Royal Society of Chemistry.

Reference

1. Ma, Y.; Mesa, C. A.; Pastor, E.; Kafizas, A.; Francas, L.; Formal, F. L.; Pendlebury, S. T.; Durrant, J. R. Rate law analysis of water oxidation and hole scavenging on a $BiVO_4$ photoanode. *ACS Energy Lett.*, 2016, **1**, 618-623.
2. Kavan, L.; Steier, L.; Grätzel, M. Ultrathin buffer layers of SnO_2 by atomic layer deposition: perfect blocking function and thermal stability. *J. Phys. Chem. C*, 2017, **121**, 342–350.
3. Selim, S.; Pastor, E.; García-Tecedor, M.; Morris, M. R.; Francas, L.; Sachs, M.; Moss, B.; Corby, S.; Mesa, C. A.; Gimenez, S.; Kafizas, A.; et al. Impact of oxygen vacancy occupancy on charge carrier dynamics in $BiVO_4$ photoanodes. *J. Am. Chem. Soc.* 2019, **141**, 47, 18791–18798.
4. Ma, Y.; Pendlebury, S. T.; Reynal, A.; Formal, F. L.; Durrant, J. R. Chem. Sci., 2014, 5, 2964–2973.
